# Physiological Effects on Coexisting Microalgae of the Allelochemicals Produced by the Bloom-Forming Cyanobacteria *Synechococcus* sp. and *Nodularia Spumigena*

**DOI:** 10.3390/toxins11120712

**Published:** 2019-12-06

**Authors:** Sylwia Śliwińska-Wilczewska, Aldo Barreiro Felpeto, Katarzyna Możdżeń, Vitor Vasconcelos, Adam Latała

**Affiliations:** 1 Division of Marine Ecosystems Functioning, Institute of Oceanography, University of Gdansk, Avenue Piłsudskiego 46, P-81-378 Gdynia, Poland; oceal@ug.edu.pl; 2 Interdisciplinary Center of Marine and Environmental Research-CIMAR/CIIMAR, University of Porto, Avenida General Norton de Matos s/n, PT-4450-208 Matosinhos, Portugal; aldo.barreiro@gmail.com (A.B.F.); vmvascon@fc.up.pt (V.V.); 3 Institute of Biology, Pedagogical University of Cracow, Podchorążych 2 St., P-30-084 Kraków, Poland; studianaturae@gmail.com; 4 Department of Biology, Faculty of Sciences, Porto University, Rua do Campo Alegre, PT-4069-007 Porto, Portugal

**Keywords:** allelopathy, algal blooms, filtrate additions, growth phase, photosynthesis, respiration, toxins

## Abstract

Only a few studies have documented the physiological effects of allelopathy from cyanobacteria against coexisting microalgae. We investigated the allelopathic ability of the bloom-forming cyanobacteria *Synechococcus* sp. and *Nodularia spumigena* filtrates on several aspects related to the physiology of the target species: population growth, cell morphology, and several indexes of photosynthesis rate and respiration. The target species were the following: two species of green algae (*Oocystis submarina*, *Chlorella vulgaris*) and two species of diatoms (*Bacillaria paxillifer*, *Skeletonema marinoi*). These four species coexist in the natural environment with the employed strains of *Synechococcus* sp. and *N. spumigena* employed. The tests were performed with single and repeated addition of cyanobacterial cell-free filtrate. We also tested the importance of the growth phase in the strength of the allelopathic effect. The negative effects of both cyanobacteria were the strongest with repeated exudates addition, and generally, *Synechococcus* sp. and *N. spumigena* were allelopathic only in the exponential growth phase. *O. submarina* was not negatively affected by *Synechococcus* filtrates in any of the parameters studied, while *C. vulgaris*, *B. paxillifer*, and *S. marinoi* were affected in several ways. *N. spumigena* was characterized by a stronger allelopathic activity than *Synechococcus* sp., showing a negative effect on all target species. The highest decline in growth, as well as the most apparent cell physical damage, was observed for the diatom *S. marinoi*. Our findings suggest that cyanobacterial allelochemicals are associated with the cell physical damage, as well as a reduced performance in respiration and photosynthesis system in the studied microalgae which cause the inhibition of the population growth. Moreover, our study has shown that some biotic factors that increase the intensity of allelopathic effects may also alter the ratio between bloom-forming cyanobacteria and some phytoplankton species that occur in the same aquatic ecosystem.

## 1. Introduction

Cyanobacteria are capable of producing a variety of metabolites for which toxicity to aquatic and terrestrial organisms has been demonstrated [1,2,3,4,5,6]. Some of these compounds might function as allelochemicals that inhibit the growth of competitors. Allelopathy is regarded as one of the factors that promote and maintain massive phytoplankton blooms in both freshwater reservoirs and oceanic habitats [7,8,9,10,11]. Therefore, more effort is needed to study the mechanisms causing cyanobacterial blooms, like, potentially, their allelopathic activity on coexisting phytoplankton species.

The mode of action of allelopathic compounds depends on the interrelationship between the different organisms, and the activity of released allelochemicals. The mechanism and exact mode of action of many of these chemical compounds are still insufficiently understood [12]. In the natural environment, the chemical functionality of allelopathic compounds is highly diverse, and donor species can affect target organisms in many different ways. In most cases, allelopathic compounds are released into the environment [13]. Allelopathic compounds secreted by cyanobacteria can inhibit photosynthesis process [14,15,16,17]. Allelochemicals may also inhibit the enzyme’s activity, RNA synthesis, and DNA replication. There were also observed changes in cell morphology, temporary cyst formation, and block of cell motility and division [13,18,19]. However, it is worth noting here that the biological role of cyanobacterial metabolites remains yet to be fully elucidated.

The goal of this work was to examine mechanisms of action, at the level of cell main physiological processes of allelochemicals from the bloom-forming cyanobacteria *Synechococcus* sp. and *N. spumigena*. Also, we studied some biotic factors that could affect the strength of allelopathy, such as specificity of donor and target organisms and growth phase of the donor species. The allelopathic ability was tested on the growth, cell morphology, photosynthetic performance, and respiration rate of a set of species of green algae (*O. submarina* and *C. vulgaris*) and diatoms (*B. paxillifer* and *S. marinoi*) that coexist with the employed strain of cyanobacteria in the natural environment.

## 2. Results

### 2.1. Effect of Cyanobacterial Exudates on Growth

**Figure 1:**
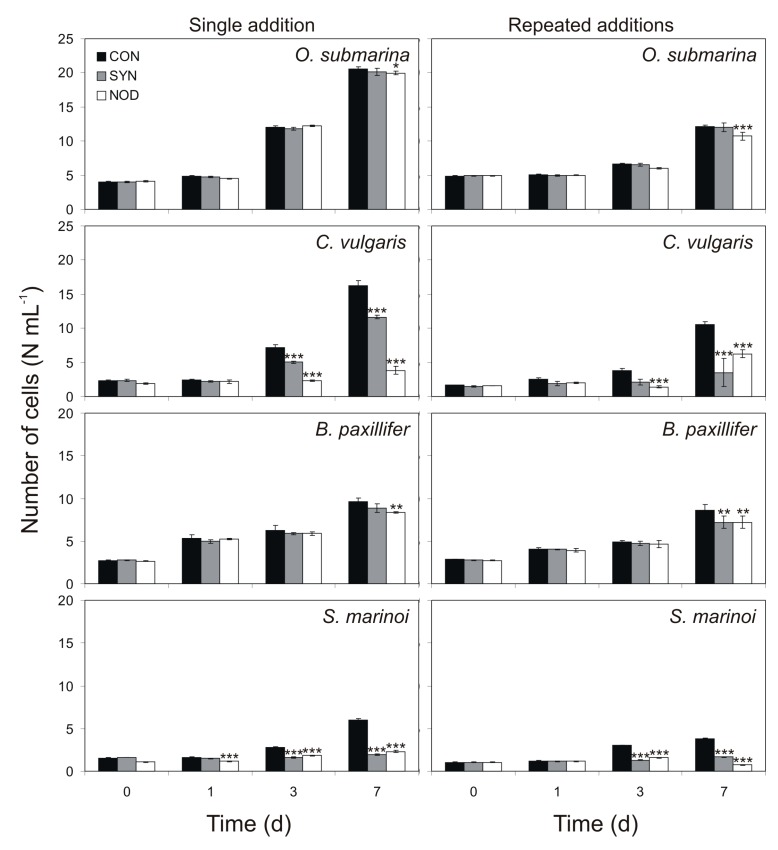
Number of cells (N) for *O. submarina* (10^5^ cell mL^−1^), *C. vulgaris* (10^6^ cell mL^−1^), *B. paxillifer* (10^5^ cell mL^−1^), and *S. marinoi* (10^6^ cell mL^−1^) for controls (CON) and treatments with single and repeated additions of exudates acquired from *Synechococcus* sp. (SYN) and *N. spumigena* (NOD) cultures in exponential growth phases (*n* = 3, mean ± SD). * signify statistically significant difference between allelopathic treatment and control (ANOVA and Tukey’s post hoc test: * *p* < 0.05; ** *p* < 0.01; *** *p* < 0.001).

The addition of exudates obtained from *Synechococcus* sp. from the exponential growth phase significantly decreased the growth of green alga *C. vulgaris*, as well as diatoms *B. paxillifer* and *S. marinoi*, but not green alga *O. submarina*. *N. spumigena*, however, showed a negative effect on all the species tested (Table 1, Figure 1).

The growth of *C. vulgaris* was also affected already on the third day by *Synechococcus* sp. filtrate, being, relative to the control, 70% (Tukey HSD test; *p* < 0.001) and 55% (Tukey HSD test; *p* < 0.001) for single and multiple exudates additions, respectively. On the seventh day, the growth in this microalga was, relative to the control, 72% (Tukey HSD test; *p* < 0.001) and 33% (Tukey HSD test; *p* < 0.001) for single and repeated filtrate addition, respectively. *B. paxillifer* growth was not so strongly inhibited as the other two species, showing a single significant effect for the repeated filtrate addition in the seventh day, being 83% relative to the control (Tukey HSD test; *p* < 0.01). The growth of *S. marinoi* was affected by *Synechococcus* sp. filtrate already at day 3, being, relative to the control, 57% (Tukey HSD test; *p* < 0.001) and 43% (Tukey HSD test; *p* < 0.001) with single and repeated additions of the filtrate, respectively. On the seventh day, the growth was reduced by 32% (Tukey HSD test; *p* < 0.001) and 44% (Tukey HSD test; *p* < 0.001) with single and repeated additions of the filtrate, respectively. *O. submarina* growth was not significantly affected by *Synechococcus* sp. filtrate.

*N. spumigena* showed a greater negative impact on tested microalgae than *Synechococcus* sp. filtrate. On the first, third, and seventh day, the growth of *S. marinoi* was reduced by 70% (Tukey HSD test; *p* < 0.001), 65% (Tukey HSD test; *p* < 0.001) and 39% (Tukey HSD test; *p* < 0.001) with single additions of filtrate, and by 52% (Tukey HSD test; *p* < 0.001) and 19% (Tukey HSD test; *p* < 0.001) with repeated exudates additions on the third and seventh day of the experiment. In *C. vulgaris*, the highest decrease in growth was observed, for the single and repeated filtrate addition, after 7 days of the experiment, being 24% and 59%, respectively, relative to the control (Tukey HSD test; *p* < 0.001 for both). In this same species, the single and repeated addition experiment showed a significant reduction of growth in the third day, for which the growth was, relative to the control, 33% (Tukey HSD test; *p* < 0.001) and 37% (Tukey HSD test; *p* < 0.001), respectively. The growth of *B. paxillifer* and *O. submarina* were affected already at the seventh day by *N. spumigena* filtrate, being, relative to the control, 87% (Tukey HSD test; *p* < 0.01) and 97% (Tukey HSD test; *p* < 0.05) for single filtrate additions and 83% (Tukey HSD test; *p* < 0.01) and 89% (Tukey HSD test; *p* < 0.001) for repeated exudates additions, respectively.

Surprisingly, the filtrate of the *Synechococcus* sp. and *N. spumigena* cultures in the stationary phase did not have a negative allelopathic effect on tested microalgae (Tukey HSD test; NS, *p* > 0.05 for all, Table 2). The only exception was the inhibition of the growth of the most sensitive species: *C. vulgaris* and *S. marinoi*. In *C. vulgaris*, for the experiment with *Synechococcus* sp. filtrate addition, the negative effect on growth was detected on the seventh day of exposure. The values were, relative to the control, 75% (Tukey HSD test; *p* < 0.001). Furthermore, the growth of *S. marinoi* was affected on the seventh day by *N. spumigena* exudates, being, relative to the control, 85% (Tukey HSD test; *p* < 0.01) for filtrate additions.

### 2.2. Allelopathic Effects of Cyanobacterial Exudates on the Cell Morphology

**Figure 2:**
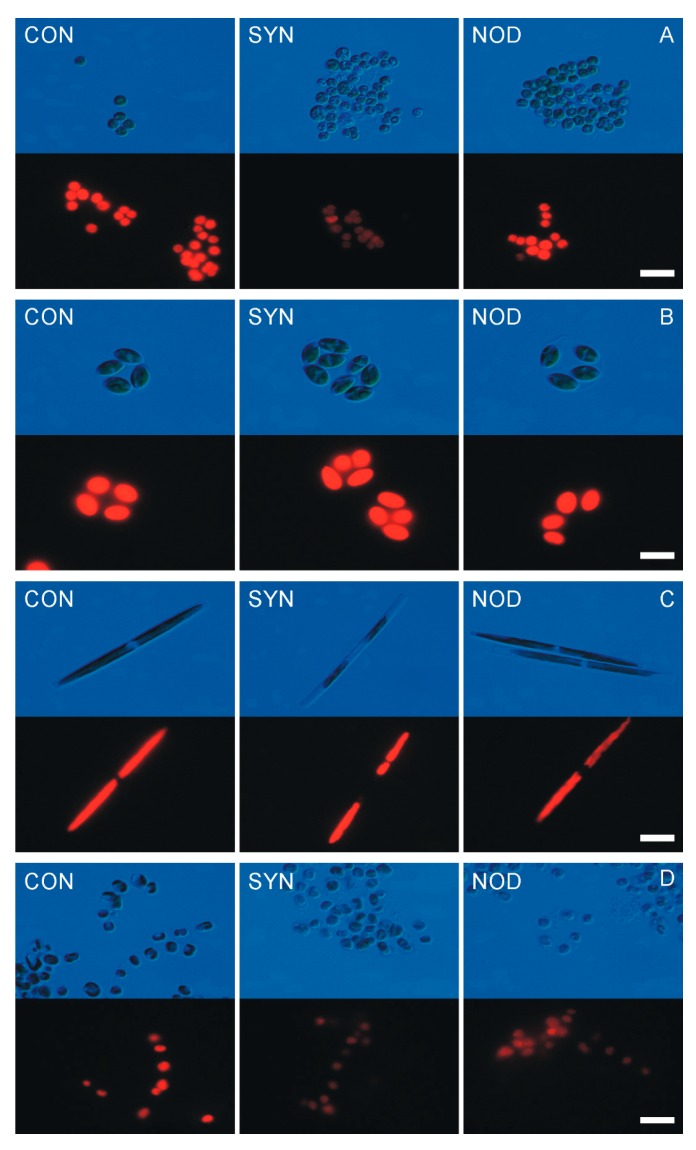
The cells morphology of *C. vulgaris* (**A**), *O. submarina* (**B**), *B. paxillifer* (**C**), and *S. marinoi* (**D**) for control (CON) and treatments with repeated additions of the exudates acquired from *Synechococcus* sp. (SYN) and *N. spumigena* (NOD) cultures in exponential growth phases after 7 days of exposure under a light microscope (top panel) and under an epifluorescence microscope (bottom panel). Scale bar = 10 µm.

The same species whose growth was the most affected by filtrates (*C. vulgaris*, *B. paxillifer*, and *S. marinoi*) also experienced abnormal morphological changes (Figure 2). Cell bleaching and deformation were observed with light microscopy, and under epifluorescence microscopy, degeneration of thylakoids. In the assay with repeated addition of filtrate, the controls of *C. vulgaris*, *B. paxillifer* and *S. marinoi* showed, on average, 98%, 96% and 96%, of healthy cells, respectively, whereas the treatment showed the proportions of 71% (Mann–Whitney test; *p* < 0.001), 84% (Mann–Whitney test; *p* < 0.001) and 64% (Mann–Whitney test; *p* < 0.001), respectively for *Synechococcus* sp. and 68% (Mann–Whitney test; *p* < 0.001), 81% (Mann–Whitney test; *p* < 0.001) and 58% (Mann–Whitney test; *p* < 0.001), respectively for *N. spumigena*. No negative effect was observed against *O. submarina*.

### 2.3. Allelopathic Effects of Cyanobacterial Exudates on P–E Curves and Photosynthetic Parameters

**Figure 3:**
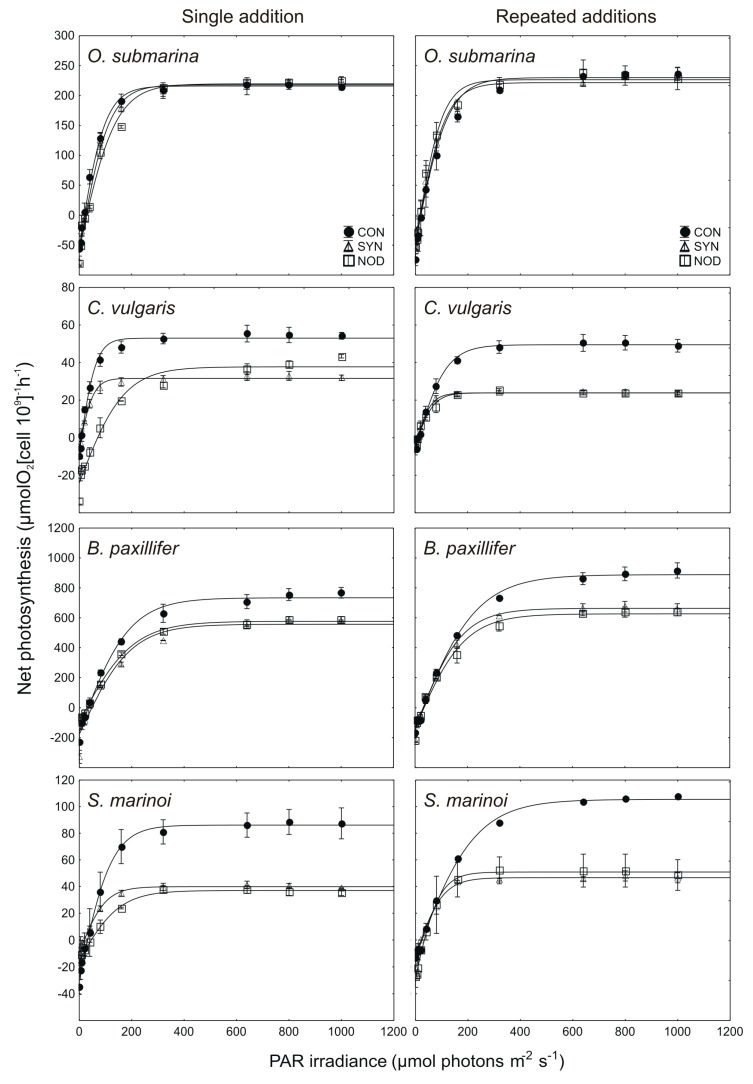
*P–E* curves for tested microalgae for controls (CON) and experiments with single and multiple additions of exudates acquired from *Synechococcus* sp. (SYN) and *N. spumigena* (NOD) cultures in exponential growth phases after 7 days of exposure (*n* = 3, mean ± SD).

**Figure 4:**
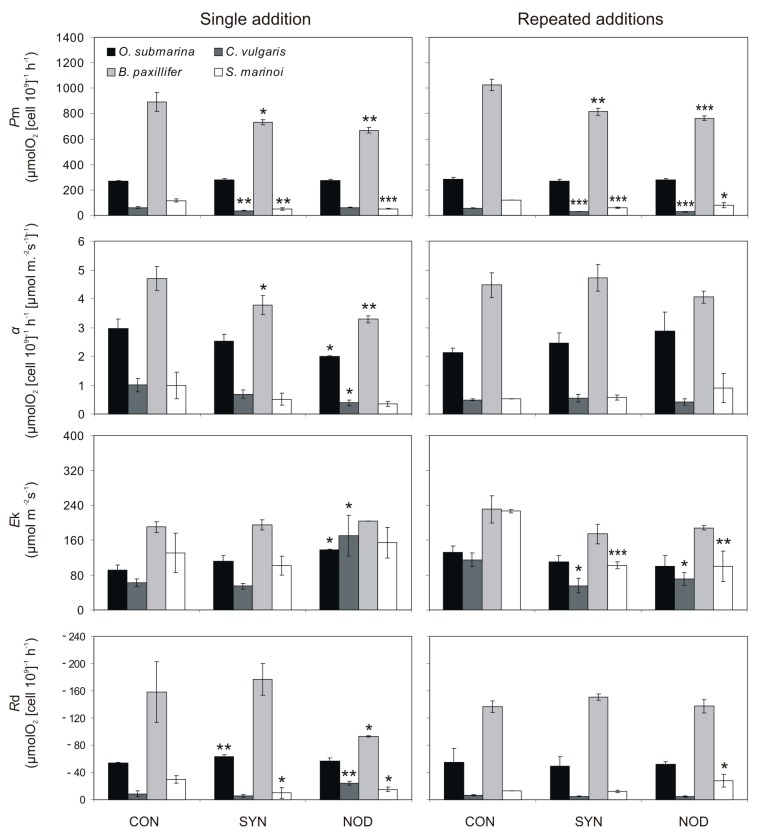
Photosynthetic parameters: *p*_m_, *a*, *E*_k_, and *R*_d_ for target species for controls (CON) and treatments with a single and multiple additions of the exudates acquired from *Synechococcus* sp. (SYN) and *N. spumigena* (NOD) cultures in exponential growth phases after 7 days of exposure (*n* = 3, mean ± SD). * signify statistically significant difference between allelopathic treatment and control (ANOVA and Tukey’s post hoc test: * *p* < 0.05; ** *p* < 0.01; *** *p* < 0.001).

The effects of single and repeated addition of exudates from cyanobacterial cultures on the *P–E* curves and photosynthetic parameters *p*_m_, *α*, *E*_k_, and *R*_d_ are shown in Figure 3 and Figure 4.

For single filtrate addition acquired from *Synechococcus* sp., the values of *p*_m_ in *C. vulgaris*, *B. paxillifer*, and *S. marinoi* constituted, respectively, 61% (*t*-test; *p* < 0.01), 83% (*t*-test; *p* < 0.05), and 44% (*t*-test; *p* < 0.01), relative to the control. Furthermore, for the *N. spumigena* filtrate the significant negative effect was found for two tested diatoms: *B. paxillifer* and *S. marinoi* in the single filtrate addition experiment, for which it constituted 75% (*t*-test; *p* > 0.01) and 45% (*t*-test; *p* > 0.001), respectively, of the control. For this same parameter, in the repeated addition experiments, the values of *p*_m_ for *C. vulgaris*, *B. paxillifer*, and *S. marinoi* were significantly different from the control by the seventh day, when they constituted, relative to the control, 52% (*t*-test; *p* < 0.001), 80% (*t*-test; *p* < 0.01), and 50% (*t*-test; *p* < 0.001), respectively for *Synechococcus* sp. exudates and 51% (*t*-test, *p* < 0.001), 75% (*t*-test; *p* < 0.001), and 67% (*t*-test; *p* < 0.05), respectively for *N. spumigena* filtrate. There was no significant effect of cyanobacterial filtrate on the *p*_m_ value of *O. submarina*, (*t*-test; NS, *p* > 0.05 for all comparisons).

Regarding the *α* parameter, for *B. paxillifer*, in the single addition assay, it was 80% relative to the control (*t*-test; *p* < 0.05). For this same species, it was not found a significant effect in the repeated addition experiment (*t*-test; NS, *p* > 0.05). For single filtrate addition acquired from *N. spumigena*, the values of *p*_m_ in *O. submarina*, *C. vulgaris*, and *B. paxillifer* constituted, respectively, 92% (*t*-test; *p* < 0.05), 42% (*t*-test; *p* < 0.05), and 70% (*t*-test; *p* < 0.01), relative to the control. There was no significant effect of repeated filtrate addition obtained from *Synechococcus* sp. and *N. spumigena* on the *α* parameter of the tested microalgal species (*t*-test, NS, *p* > 0.05).

The single addition of *Synechococcus* sp. exudates did not cause a significant effect in the parameter *E*_k_ for any of the species tested (*t*-test; NS, *p* > 0.05, for all comparisons). However, for single filtrate addition acquired from *N. spumigena*, the values of *E*_k_ in *O. submarina* and *C. vulgaris* constituted, respectively, 102% (*t*-test; *p* < 0.05) and 280% (*t*-test; *p* < 0.05), relative to the control. In the repeated exudates addition assay, the *E*_k_ values of *C. vulgaris* and *S. marinoi* were, relative to the control 49% (*t*-test; *p* < 0.05) and 45% (*t*-test; *p* < 0.001), respectively for *Synechococcus* sp. exudates and 63% (*t*-test; *p* < 0.05) and 44% (*t*-test; *p* < 0.01), respectively, for *N. spumigena* filtrate. There was no significant effect of repeated filtrate addition acquired from *Synechococcus* sp. and *N. spumigena* on the *E*_k_ of the *O. submarina* and *B. paxillifer* (*t*-test; NS, *p* > 0.05 for all).

For the dark respiration (parameter *R*_d_) the only significant negative effect of *Synechococcus* sp. filtrate was found for *S. marinoi* in the single exudate addition assay, for which it constituted 38% of the control (*t*-test; *p* < 0.05). In the same single filtrate addition experiment, in *O. submarina*, the effect was significantly positive, being 17% higher than the control (*t*-test; *p* < 0.01). Repeated addition of *Synechococcus* sp. exudates did not cause a significant effect in the parameter *R*_d_ for any of the species tested (*t*-test; NS, *p* > 0.05, for all comparisons). Regarding the *R*_d_ parameter, for the diatoms *B. paxillifer* and *S. marinoi*, in the single addition experiment with exudates from *N. spumigena*, the reduction was 62% (*t*-test; *p* < 0.05) and 55% (*t*-test; *p* < 0.05) relative to the control. In the same experiment, in *C. vulgaris*, the effect was significantly positive, being 264% higher than the control (*t*-test; *p* < 0.01). A significant effect of *N. spumigena* in the multiple exudates addition treatment was found only for *S. marinoi*, for which it constituted 217% of the control (*t*-test; *p* < 0.05).

## 3. Discussion

### 3.1. Effect of the Selection of the Donor and Target Organisms

In the present work, we showed how several coexisting microalgae responded differently to cyanobacterial allelopathy. Coevolution and specific traits (cell wall thickness, surface-area relationship, etc.) are hypothesized to be the main reasons explaining the differences in susceptibility to allelochemicals [20]. These differences in susceptibility are found between major taxonomic groups, but also within them [21,22,23]. This differential effect on microalgae species suggests that cyanobacterial allelochemicals could have an important role in structuring phytoplankton communities.

We detected that population growth was the most negatively affected variable in the green alga *Chlorella vulgaris* and the diatom *Skeletonema marinoi*. Previous studies also reported detrimental allelopathic impact of cyanobacteria on green algae [16,24,25,26,27] and diatoms [17,20,27,28]. However, some species of green algae co-occurred with toxic and bloom-forming cyanobacteria during the summer period in the Baltic Sea [29,30,31]. Therefore, resistance to *Synechococcus* sp. and *N. spumigena* allelochemicals may differ between species within this group. The susceptibility of *C. vulgaris* and *S. marinoi* could be a consequence of its small cell size (less than 5 µm), and hence, relatively large surface to volume relationship, which enhances the efficiency of uptake of extracellular molecules [32]. Conversely, *Synechococcus* sp. exudates had no allelopathic impact on the abundance of *Oocystis submarina*. This could be due, at least in part, to the formation of colonies with thin, hyaline mucilaginous envelope. However, *N. spumigena* was characterized by a stronger allelopathic activity than *Synechococcus* sp., showing a negative effect on all tested species. This may be due to the release of higher amounts of allelochemicals or more potent compounds than those secreted by unicellular picocyanobacterium *Synechococcus* sp.

### 3.2. Effect of Single and Repeated Addition of Cyanobacterial Exudates

A greater amount of allelopathic compounds present in the filtrate should have a stronger effect on target organisms [20,33,34]. We also showed that negative effects against the population growth of tested microalgae were amplified by multiple exudates additions. These findings could be explained simply by the renewal of cyanobacterial allelochemicals, which naturally degrade in several ways, like bacterial activity or light [33,35].

We examined the allelopathic ability of *Synechococcus* sp. and *N. spumigena* using non-axenic cultures. Heterotrophic bacteria may mediate allelopathic interactions between different species by altering cyanobacterial allelochemicals or releasing chemical substances on their own [36]. However, Suikkanen et al. [20] revealed that exudates containing only allelochemicals released by heterotrophic bacteria did not affect target microalgae species, which would reduce the potential influence of accompanying bacteria in our work.

### 3.3. Comparison of Cyanobacterial Cell-Free Filtrate in Exponential and Stationary Growth Phases

A relevant biotic factor influencing allelopathic interactions is the growth phase of the donor organism. The release of allelopathic compounds by donor organisms could depend on their growth phase [7]. In this work, we demonstrated that *Synechococcus* sp. and *N. spumigena* were more allelopathic in exponential than in the stationary growth phase. Suikkanen et al. [20] and Kubanek et al. [7] also showed that the culture exudates acquired from *N. spumigena* and *K. brevis*, respectively, from the exponential phase of growth, had a detrimental allelopathic effect, whereas the exudates from stationary phase did not show this same effect. Schmidt and Hansen [37] concluded that cultures in the exponential growth phase exhibited greater allelopathic effects than cultures in stationary phase. However, this finding is not generalizable to all allelopathic phytoplankton species [38]. Authors that observed a greater effect for cultures from the exponential growth phase, suggested that allelopathic activity could be more related to the production of a complex of allelopathic compounds than to specific, single substances, i.e., toxins [39].

### 3.4. Mode of Action of Allelochemicals

Among the allelopathic effects detected in our study, there was physical damage to cell structures in three species, as observed by light and epifluorescence microscope. Other authors have described as well morphological changes or physical damage to cells caused by allelopathic activity [40,41,42].

Population growth and physical damage to cell structures could be end-of-chain (distal) effects. As a consequence of allelochemicals effects at the metabolic or physiological scale (proximal), general cell processes would be negatively affected, and the ultimate consequence could be physical cell damage and growth inhibition. However, it is also possible that cell physical damage was the proximal effect of allelochemicals, and then would come improper functioning of metabolism and physiological processes. This would be the case if allelochemicals were some sort of cytolytic compounds, not rare among phytoplankton [43,44].

We also tested the photosynthetic and dark respiration rates, to evaluate the effects on physiological processes in more detail. The inhibition of photosynthesis via the secretion of allelopathic compounds may be another effective strategy for cyanobacteria, as demonstrated by Ma et al. [16]. It is worth noting that the reduced performance of respiration and PS system might be due to the indirect consequence of the allelopathic mediation rather than a direct effect of secreted metabolites. In this work, we showed that the photosynthetic parameters of our tested organisms were significantly altered. *p*_m_ was shown to be the most sensitive parameter to detect allelopathic effects. The parameter *E*_k_ is a photosynthetic characteristic that is independent of algal biomass [45,46]. Moreover, Light and Beardall [46] suggested that reduction of both the *E*_k_ and *p*_m_ values, and a minimal change in *α*, indicates that agents other than main pigment concentration are impacting the photosynthetic performance of light-harvesting and energy transduction in microalgae. In our work, we assumed that this factor could be cyanobacterial allelopathy. Besides, the *α* parameter was also significantly reduced. The *α* parameter is the maximum light utilization (assimilation of CO_2_) coefficient. Allelochemicals may inhibit the CO_2_ assimilation rate by modifying the stomata function, as suggested by Zhou and Yu [47]. Much of the early work on the allelochemical modes of action considered their effects on respiration [48]. In this work, both a decrease and an increase in *R*_d_ parameters were observed. Respiration process can be directly disadvantaged through many modes of action. Secondary metabolites can both uncouple oxidative phosphorylation and inhibit mitochondrial electron transport.

One of the most unfinished tasks in the study of allelopathy is to identify the compounds responsible for the observed effects, where only a few works exist that have resolved this issue [2]. Cyanobacterial toxins have been thoroughly studied from the perspective of several fields of biology, but our study showed that non-toxic strains are also able to secrete compounds that inhibit co-occurring species. Our findings suggest that cyanobacterial allelochemicals are associated with the cell physical damage, as well as a reduced performance in respiration and photosynthesis system in the studied microalgae which cause the inhibition of the population growth. Moreover, our study has shown that some biotic factors such as specificity of donor and target organisms and growth phase of the donor species, that increase the intensity of allelopathic effects may also alter the ratio between bloom-forming cyanobacteria and some phytoplankton species that occur in the same aquatic ecosystem. The exact compounds involved in the observed effects were not studied and it remains to be investigated whether the observed effects result from the action of one particular metabolite or rather a mixture of different compounds. The identification of chemical structures of cyanobacterial metabolites and further studies focusing only on the isolated compounds are important steps toward elucidating their biological role.

## 4. Materials and Methods

### 4.1. Culture Conditions

The strains of *Synechococcus* sp. and *Nodularia spumigena* employed were CCBA-124 and CCBA-15, respectively, and the target microalgae were the green algae *Oocystis submarina* CCBA-01 and *Chlorella vulgaris* CCBA-80, and the diatoms *Bacillaria paxillifer* CCBA-14 and *Skeletonema marinoi* CCBA-98. All these strains were isolated from the Baltic Sea and were maintained as unispecies cultures in the Culture Collection of Baltic Algae (CCBA).

These strains were grown in f/2 culture medium [49] in 25-mL glass Erlenmeyer flasks that were stirred daily during the experiment. The f/2 medium was performed with Baltic Sea water filtered through glass fiber membranes (Whatman GF/C, Saint Louis, MO, USA) and autoclaved. Salinity was 8 PSU, measured with salinometer (inoLab Cond Level 1, Weilheim in Oberbayern, Germany). Cyanobacterial cultures were kept in a culture room at 20 °C with a 16:8 h light:dark cycle. The intensity of photosynthetically sctive radiation (PAR) was 190 μmol photons m^−2^s^−1^, measured with a quantum-meter (LI-COR; Lincoln, NE, USA) with a cosine collector. All the target cultures employed in the experiments were kept under the same conditions at least 7 days prior to the experiments.

### 4.2. Cell-Free Filtrate Experiments

Allelopathic effects were tested in conformity with the methods from Śliwińska-Wilczewska and Latała [27] with certain modifications. Allelopathic ability was identified by adding exudates acquired from donor cyanobacteria cultures to the target green algae and diatom species. The donor *Synechococcus* sp. and *N. spumigena* cultures were gently filtered using a 0.45-µm filter (Macherey-Nagel MN GF-5; Düren, Germany). The cell abundance in the donor cultures was about 10^7^ mL^−1^ for *Synechococcus* sp. and 10^6^ mL^−1^ for *N. spumigena*. Prior to the experiments, the concentrations of nutrients (N-NO_3_ and P-PO_4_) in the cyanobacterial cultures were measured using DR6000 Spectrophotometer (Hach-Lange, Loveland, CO, USA) according to Grasshoff [50]. Then, the concentrations of major nutrients in the controls and allelopathic assays were adjusted to the same level as in the f/2 medium. The filtrates obtained were analyzed on an epifluorescence microscope (Nikon Eclipse 80i; Tokyo, Japan) to verify the absence of donor cyanobacteria cells.

We performed two kind of experiments: (a) single addition (at the beginning of the experiments) of cell-free culture filtrate from the cyanobacteria; and (b) repeated addition (daily) of culture filtrate. The last experiments were aimed to simulate continuous release of allelochemicals. Treatments were executed by adding the cell-free filtrate (V = 2 mL) to 25-mL Erlenmeyer flasks containing cell suspensions of the target species (V = 20 mL). Controls consisted in the addition of filtered f/2 medium (V = 2 mL) to 25-mL Erlenmeyer flasks containing cell suspensions of the same microalgae species (V = 20 mL). Separate treatments were prepared with exudates from cyanobacterial cultures in exponential and stationary phases of growth. *Synechococcus* sp. and *N. spumigena* cultures from exponential growth phase were kept in a culture room at 8 PSU, 20 °C and 190 μmol photons m^−2^s^−1^ with a 16:8 h light:dark cycle for one week, while cyanobacterial cultures from stationary growth phases were kept in the same conditions for three weeks. The cyanobacterial growth phases were determined by monitoring cell abundances. In the repeated filtrate addition experiments, the filtrate was daily renewed by replacing 2-mL of cell suspension from the experimental flasks with an equal volume of fresh filtrate (for the experimental assay) or filtered f/2 medium (for the controls). All the experimental treatments had three replicates. The initial concentration of the experimental cultures was 0.8 µg Chl *a* mL^−1^. In the case of cultures obtained from the stationary growth phase, the *Synechococcus* sp. and *N. spumigena* were first diluted with the f/2 medium to obtain a suitable concentration of Chl *a* and then filtered to obtain a cell-free filtrate. This concentration was low enough to represent relevant environmental conditions and high enough to be measured properly. The experimental duration was 7 days.

### 4.3. Examination of Cell Abundances

Cell abundances were assessed by measuring optical density (OD) at 750 nm with a Multiskan GO spectrophotometer (Thermo Scientific, Waltham, MA, USA) and then transforming these values to cell abundances with a previously fitted linear regression model between cell abundance (N mL^−1^) and optical density (OD). In order to fit this model, cyanobacterial and microalgal cells were counted under the microscope in a Bürker counting chamber [51]. OD was measured in the same samples, and these data were used to fit a linear regression model between the variables N mL^−1^ and OD. The linear regression models for *Synechococcus* sp. and *N. spumigena* were chosen based on the model proposed by Barreiro Felpeto et al. [19]. The linear regression models for tested microalgae were described in Śliwińska-Wilczewska and Latała [27]. Cell abundances in the experiments were measured at times zeroth (1h) and first, third, and seventh day of the allelopathic assay.

### 4.4. Examination of Cell Morphology

Cell morphology of target species was examined in samples from the seventh day of the experiment with repeated additions of cell-free filtrate. The analysis followed the method from Barreiro Felpeto et al. [19]. The samples were determined using a microscope (Nikon Eclipse 80i, Tokyo, Japan). It would be best to observe the same cell under light and epifluorescence microscope. It is, however, not possible to do so as using epifluorescence microscopy requires filtering the material on the polycarbonate filter (Whaman Nuclepore^™^ Track-Etched Membranes, Saint Louis, MO, USA) and therefore, the same single cell cannot be viewed under a light microscope.

### 4.5. Measurements of the Rate of Photosynthesis

Oxygen production of target species were examined in the experiments with single and repeated additions of *Synechococcus* sp. and *N. spumigena* cell-free filtrate in exponential growth phases. Measurements of oxygen production were carried out on the seventh day of the experiment with a Clark-type oxygen electrode (Hansatech Chlorolab 2, King’s Lynn, UK). Experimental data were fitted to the photosynthesis irradiance response curves (*P–E* curves) using the equation from Jassby and Platt [52] with software Statistica® 13.1 (StatSoft Polska, Kraków, Poland). Following Sakshaug et al. [53], the parameters estimated were: photosynthetic capacity—*p*_m_, maximum light utilization coefficient—*α*, light saturation index—*E*_k_, and dark respiration—*R*_d_.

### 4.6. Statistical Analyses

Repeated measures ANOVA, with time (days) as covariate, was used to test the effect of cyanobacterial filtrate on growth of the tested microalgae. A post-hoc Tukey’s HSD test was used to determine significant differences between control and allelopathic assay. Differences in the abundance of the recognized morphological types of cells were analyzed with a Mann–Whitney test. The *t*-tests were used to determine whether the photosynthetic parameters of the target microalgae species, when treated with cyanobacterial exudates, differed from the control on the last day of the allelopathic assay. Data are showed as means ± standard deviations (SD). Levels of significance were: * *p* < 0.05; ** *p* < 0.01; *** *p* < 0.001. The statistical analyses were executed using Statistica® 13.1. (StatSoft Polska, Kraków, Poland).

## Figures and Tables

**Table 1:** Results of two-way factorial ANOVA for single and repeated additions of exudates acquired from cyanobacteria cultures in exponential growth phases (Df—degrees of freedom; Ss—sum of squares; Mss—mean sum of squares; F—Fisher's F-test statistic; * *p* < 0.05; ** *p* < 0.01; *** *p* < 0.001).

Response Variable (Number of Cells)	Source of Variation	Df	Ss	Mss	F	Ss	Mss	F
			*Synechococcus* sp.			*N. spumigena*		
			Single Additions of Cell-Free Filtrate					
*O. submarina*	filtrate	1	0.184	0.184	2.896	0.163	0.163	4.595 *
	time	3	1039.170	346.390	5459.262 ***	1037.309	345.770	9726.294 ***
	filtrate time *	3	0.135	0.045	0.708	0.543	0.181	5.090 *
	error	16	1.015	0.063		0.569	0.036	
*C. vulgaris*	filtrate	1	17.839	17.839	138.156 ***	119.410	119.410	721.685 ***
	time	3	535.876	178.625	1383.420 ***	242.597	80.866	488.730 ***
	filtrate time *	3	20.064	6.688	51.798 ***	144.634	48.211	291.376 ***
	error	16	2.066	0.129		2.647	0.165	
*B. paxillifer*	filtrate	1	0.813	0.813	6.688 *	1.216	1.216	13.770 **
	time	3	129.403	43.134	355.008 ***	119.790	39.930	452.322 ***
	filtrate time *	3	0.409	0.136	1.123	1.346	0.449	5.083 *
	error	16	1.944	0.122		1.412	0.088	
*S. marinoi*	filtrate	1	10.588	10.588	1341.763 ***	11.724	11.724	1517.087 ***
	time	3	22.632	7.544	955.952 ***	31.078	10.359	1340.444 ***
	filtrate time *	3	15.964	5.321	674.305 ***	10.315	3.438	444.896 ***
	error	16	0.126	0.008		0.124	0.008	
			**Repeated Additions of Cell-Free Filtrate**					
*O. submarina*	filtrate	1	0.029	0.029	0.387	1.500	1.500	26.882 ***
	time	3	204.989	68.330	898.485 ***	170.116	56.705	1016.222 ***
	filtrate time *	3	0.025	0.008	0.108	1.844	0.615	11.018 ***
	error	16	1.217	0.076		0.893	0.056	
*C. vulgaris*	filtrate	1	34.016	34.016	56.588 ***	19.960	19.960	238.473 ***
	time	3	109.672	36.557	60.816 ***	178.845	59.615	712.265 ***
	filtrate time *	3	45.661	15.220	25.320 ***	17.262	5.754	68.748 ***
	error	16	9.618	0.601		1.339	0.084	
*B. paxillifer*	filtrate	1	1.096	1.096	8.723 **	1.456	1.456	10.204 **
	time	3	85.712	28.571	227.319 ***	86.871	28.957	202.981 ***
	filtrate time *	3	2.009	0.670	5.328 *	1.764	0.588	4.121 *
	error	16	2.011	0.126		2.283	0.143	
*S. marinoi*	filtrate	1	5.750	5.750	2175.294 ***	7.915	7.915	3161.742 ***
	time	3	11.730	3.910	1479.173 ***	8.253	2.751	1098.877 ***
	filtrate time *	3	5.618	1.873	708.431***	9.551	3.184	1271.713 ***
	error	16	0.042	0.003		0.040	0.003	

**Table 2:** Effects of stationary phase culture filtrates of *Synechococcus* sp. and *N. spumigena* on target species, expressed as the number of cells (N): *O. submarina, B. paxillifer*—10^5^ cell mL^−1^; *C. vulgaris, S. marinoi*—10^6^ cell mL^−1^, and the percentage of intact cells in the treated microalgal cultures in relation to the control (*n* = 3, mean ± SD) and results of two-way factorial ANOVA for additions of exudates acquired from cyanobacterial cultures (Df—degrees of freedom; Ss—sum of squares; Mss —mean sum of squares; F—Fisher's F-test statistic; * *p* < 0.05; ** *p* < 0.01; *** *p* < 0.001).

Target Species	Day	Number of Cells (N)	% of Control	Source of Variation	Df	Ss	Mss	F
***Synechococcus* sp.**								
*O. submarina*	0	0.619 ± 0.007	100.3 ± 1.5	filtrate	1	0.004	0.004	5.045 *
	1	0.649 ± 0.015	98.2 ± 1.6	time	3	1.571	0.524	651.147 ***
	3	0.815 ± 0.032	97.1 ± 2.5	filtrate time *	3	0.004	0.001	1.816
	7	1.227 ± 0.054	94.6 ± 1.9	error	16	0.013	0.001	
*C. vulgaris*	0	2.635 ± 0.098	99.9 ± 9.6	filtrate	1	2.885	2.885	15.230 **
	1	2.545 ± 0.061	96.4 ± 12.3	time	3	78.566	26.189	138.273 ***
	3	3.692 ± 0.205	88.4 ± 5.4	filtrate time *	3	4.282	1.427	7.536 **
	7	5.994 ± 0.247	74.7 ± 11.3	error	16	3.030	0.189	
*B. paxillifer*	0	0.267 ± 0.012	99.1 ± 2.7	filtrate	1	0.001	0.001	1.809
	1	0.254 ± 0.002	87.9 ± 2.3	time	3	0.312	0.104	359.793 ***
	3	0.405 ± 0.022	101.1 ± 3.6	filtrate time *	3	0.001	0.000	1.605
	7	0.543 ± 0.036	99.1 ± 5.0	error	16	0.005	0.000	
*S. marinoi*	0	1.379 ± 0.063	99.8 ± 2.4	filtrate	1	0.081	0.081	4.915 *
	1	1.757 ± 0.099	97.4 ± 4.2	time	3	68.187	22.729	1380.932 ***
	3	2.822 ± 0.062	98.6 ± 4.6	filtrate time *	3	0.129	0.043	2.621
	7	5.508 ± 0.205	93.7 ± 2.4	error	16	0.263	0.016	
***N. spumigena***								
*O. submarina*	0	0.621 ± 0.009	100.7 ± 1.1	filtrate	1	0.002	0.002	2.496
	1	0.661 ± 0.009	100.0 ± 0.9	time	3	1.623	0.541	562.660 ***
	3	0.799 ± 0.030	95.2 ± 2.3	filtrate time *	3	0.003	0.001	1.015
	7	1.253 ± 0.067	96.6 ± 3.5	error	16	0.015	0.001	
*C. vulgaris*	0	1.903 ± 0.073	94.6 ± 8.5	filtrate	1	1.157	1.157	10.760 **
	1	2.179 ± 0.028	90.3 ± 13.9	time	3	32.580	10.860	100.966 ***
	3	2.391 ± 0.112	91.9 ± 4.4	filtrate time *	3	1.041	0.347	3.226
	7	4.335 ± 0.609	79.8 ± 16.2	error	16	1.721	0.108	
*B. paxillifer*	0	0.273 ± 0.012	101.2 ± 2.5	filtrate	1	0.003	0.003	13.622 **
	1	0.254 ± 0.005	87.9 ± 1.5	time	3	0.262	0.087	350.093 ***
	3	0.386 ± 0.026	96.3 ± 4.0	filtrate time *	3	0.002	0.001	3.151
	7	0.499 ± 0.027	91.1 ± 3.6	error	16	0.004	0.000	
*S. marinoi*	0	1.083 ± 0.016	104.0 ± 1.4	filtrate	1	0.086	0.086	9.304 **
	1	1.192 ± 0.016	100.0 ± 0.0	time	3	8.020	2.673	290.170 ***
	3	1.825 ± 0.008	95.0 ± 0.0	filtrate time *	3	0.199	0.066	7.218 **
	7	2.294 ± 0.083	84.7 ± 5.4	error	16	0.147	0.009	
